# The Expression and Function of Fatty Acid Transport Protein-2 and -4 in the Murine Placenta

**DOI:** 10.1371/journal.pone.0025865

**Published:** 2011-10-20

**Authors:** Takuya Mishima, Jeffrey H. Miner, Mayumi Morizane, Andreas Stahl, Yoel Sadovsky

**Affiliations:** 1 Department of Obstetrics, Gynecology and Reproductive Sciences, Magee-Womens Research Institute, University of Pittsburgh, Pittsburgh, Pennsylvania, United States of America; 2 Departments of Internal Medicine, Cell Biology and Physiology, Washington University, St. Louis, Missouri, United States of America; 3 Department of Nutritional Sciences and Toxicology, University of California, Berkeley, California, United States of America; 4 Department of Microbiology and Molecular Genetics, University of Pittsburgh, Pittsburgh, Pennsylvania, United States of America; Institute of Zoology, Chinese Academy of Sciences, China

## Abstract

**Background:**

The uptake and trans-placental trafficking of fatty acids from the maternal blood into the fetal circulation are essential for embryonic development, and involve several families of proteins. Fatty acid transport proteins (FATPs) uniquely transport fatty acids into cells. We surmised that placental FATPs are germane for fetal growth, and are regulated during hypoxic stress, which is associated with reduced fat supply to the fetus.

**Methodology/Principal Findings:**

Using cultured primary term human trophoblasts we found that *FATP2*, *FATP4* and *FATP6* were highly expressed in trophoblasts. Hypoxia enhanced the expression of trophoblastic *FATP2* and reduced the expression of *FATP4*, with no change in *FATP6*. We also found that *Fatp2* and *Fatp4* are expressed in the mouse amnion and placenta, respectively. Mice deficient in *Fatp2* or *Fatp4* did not deviate from normal Mendelian distribution, with both embryos and placentas exhibiting normal weight and morphology, triglyceride content, and expression of genes related to fatty acid mobilization.

**Conclusions/Significance:**

We conclude that even though hypoxia regulates the expression of *FATP2* and *FATP4* in human trophoblasts, mouse *Fatp2* and *Fatp4* are not essential for intrauterine fetal growth.

## Introduction

Both the human and mouse placenta are hemochorial, with fetal-derived trophoblasts bathed in maternal blood, and are thus well-positioned to regulate placental transport functions, including transport of oxygen, nutrients, and waste products between the maternal and fetal blood. Among transported nutrients, the uptake and trafficking of fatty acids is critical for embryonic development and growth in all eutherians, particularly during the second half of pregnancy, when the fetal/placental growth ratio is markedly increased, corresponding to increasing fetal caloric demands [1]–[3]. Transported essential fatty acids (linoleic acid, and α-linolenic acid) are metabolized into long chain poly-unsaturated fatty acids (LCPUFAs), and are necessary for development of vital organs such as the heart and lung. A particularly high amount of arachidonic acid and docosahexaenoic acid is needed for development of the brain and retina [3]–[8]. Fatty acids are also essential for biosynthesis of membrane phospholipids, myelin, gangliosides, glycolipids and sphingolipids, and for production of signaling eicosanoids [9]–[12]. Albumin-bound free fatty acids (FFA), VLDL, and chylomicrons in the maternal circulation are the major source of fatty acids to the placenta, and require the action of trophoblastic triglyceride hydrolase for liberation of FFA and transport across the trophoblastic microvillous membrane [13]–[15]. The mechanisms underlying trophoblast fatty acid uptake and trafficking are largely unknown. Membrane-bound and cytoplasmic fatty acid binding proteins (FABPs) are expressed in trophoblasts, but their function in intracellular trafficking of fatty acids in trophoblasts is unknown [16]–[18].

Cytoplasmic FFAs bound to fatty acid binding proteins (FABPs) are targeted for metabolism or storage in lipid droplets, which are dynamic organelles that actively store neutral lipids (such as triglycerides, cholesteryl esters and retinol esters) [19]–[21]. In addition to their neutral fats, lipid droplets are encased within a layer of amphipathic lipids, and coated by lipid droplet-associated (PLIN) proteins that regulate the assembly, maintenance, and composition of lipid droplets, as well as lipolysis and lipid efflux [22]–[25].

The family of fatty acid transport proteins (FATPs, solute carrier family 27, SLC27) is an evolutionarily conserved group of integral trans-membrane proteins which, along with fatty acid translocase (FAT/CD36), mediate cellular uptake of long-chain and very long chain fatty acids. This prevalent, saturable, carrier-regulated process is distinct from the less common, passive (“flip-flop”) membrane diffusion [26]–[28]. FATPs comprise a family of six highly homologous proteins, which are expressed primarily in fatty acid-utilizing tissues [28]–[30]. Interestingly, FATP4 is also highly expressed by epithelial cells of the visceral endoderm and localizes to the brush-border of extraembryonic endodermal cells [31]. It is hypothesized that FATP1, FATP2, and FATP4 are bifunctional, exhibiting both transport and acyl-CoA synthase activities, which facilitate fatty acid influx across biological membranes [32]–[34].

The expression of placental FATPs and their regulation in this tissue is largely unknown. We recently showed that ligand-stimulated PPARg enhances the expression of FATP1 and FATP4 as well as PLIN2 in primary human trophoblast (PHT) [35], and that hypoxic trophoblasts retain neutral lipids in the form of lipid droplets (35, and manuscript in preparation). In this study we sought to identify key FATPs that are expressed in the human placenta and regulated during hypoxic stress, and use *Fatp* mutant mice to decipher the function of relevant FATPs *in vivo*.

## Results

**Figure 1:**
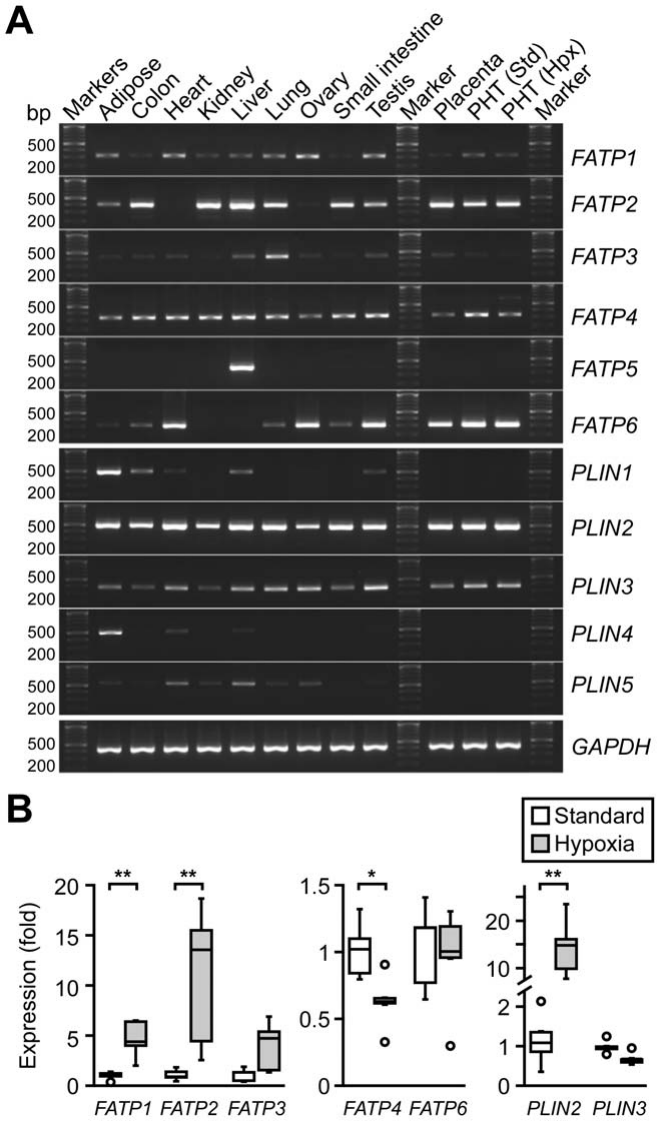
FATP and PLINs transcripts expression in PHT cells, human placentas and other tissues. (A) Expression of *FATP1-6* and *PLIN1-5* in diverse human tissues and PHT cells in standard or hypoxic conditions. Transcripts were detected by standard RT-PCR (representative results, n = 3). (B) RT-qPCR analysis of *FATP1*, *2*, *3*, *4, 6* and *PLIN2* and *3* in standard *vs.* hypoxic PHT cells (n = 5). * denotes p<0.05, ** denotes p<0.01.

We initially examined the expression of *FATP* transcripts in the human placenta and in isolated primary term trophoblasts (PHTs), and compared the level of *FATP* expression to that of other human tissues, serving as controls. Because hypoxia increases the accumulation of lipid droplets in trophoblasts [35], we also assessed the expression of *FATPs* in hypoxic PHT cells. As shown in Fig. 1A, *FATP2*, *FATP4*, and *FATP6* are clearly expressed in the placenta and in PHT cells, with weaker expression of *FATP1* and *FATP3*. *FATP5* is not expressed in the human placenta. As control, we also assessed the expression of the lipid droplet–associated (*PLIN*) transcripts, and detected the expression of *PLIN2* and *PLIN3*, but not the other members of this family. Among the highly expressed *FATPs*, we found that hypoxia enhanced the expression of *FATP2*, but diminished the expression of *FATP4*, with no change in *FATP6* (Fig. 1B). Notably, the weakly expressed *FATP1* was also increased in hypoxia. The increase in *PLIN2* in hypoxic PHTs (Fig. 1B) was expected [36], and suggests lipid droplet accumulation in hypoxic PHT cells. Together, these data indicate that placental *FATP2* and *FATP4* are relatively highly expressed in primary human trophoblasts, and that hypoxia has an opposite influence on their expression in human trophoblasts. Therefore, our subsequent analysis centered on FATP2 and FATP4.

**Figure 2:**
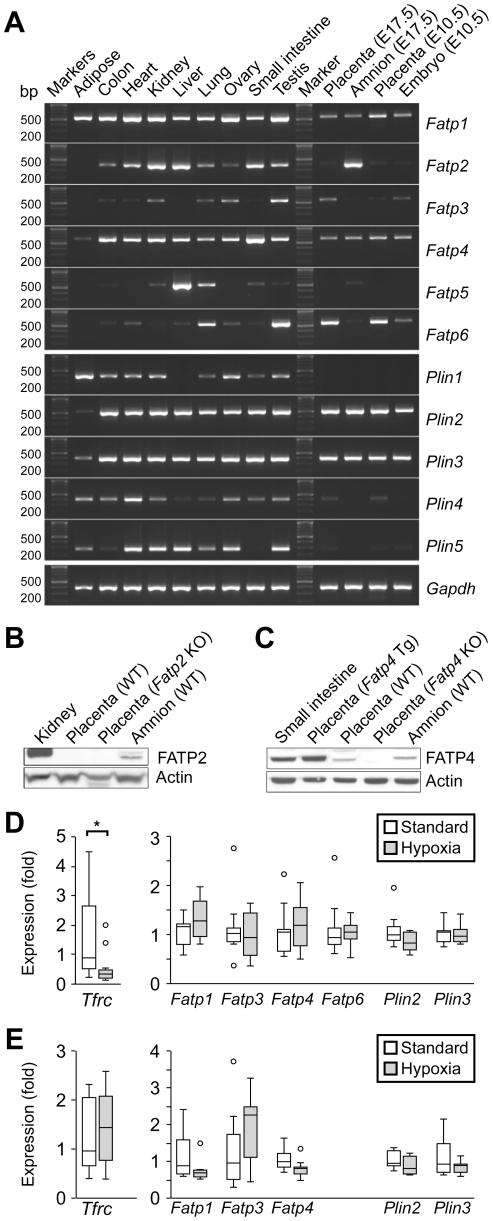
Fatp and Plin transcript expression in mouse placentas and other tissues. (A) Expression of *Fatp1-6*, *Plin1-5* in diverse mouse tissues and in the placenta (E17.5), amnion (E17.5), placenta (E10.5), and embryo (E10.5), detected by standard RT-PCR (representative results, n = 3). (B) Western blot analysis of FATP2. (C) Western blot analysis of FATP4. (D) RT-qPCR of Tfrc, *Fatp1*, *3*, *4*, *6,* and *Plin2*, *3* in mouse placentas under standard *vs.* hypoxic conditions (n = 8). (E) RT-qPCR of Tfrc, *Fatp1*, *2*, *4*, and *Plin2*, *3* in mouse amnion under standard *vs.* hypoxic conditions (n = 10). * denotes p<0.05.

To gain insight into the role of placental FATP2 and FATP4 *in vivo*, we initially sought to examine the expression of *Fatps* in placentas of wild type C57Bl/6 mice. We found several differences in *Fatp* expression between human and murine placentas. The near term mouse placenta expresses primarily *Fatp1*, *Fatp3*, *Fatp4*, and *Fatp6* (Fig. 2A). Interestingly, *Fatp2* is expressed mainly in the mouse amnion, but not in the placenta (Fig. 2A). We confirmed the expression pattern of FATP2 and FATP4 proteins in the placenta and amnion (Fig. 2B–C).

The expression of *Fatps* in other mouse organs was similar, but not identical, to that in humans (compare Fig. 1A–2A). The expression pattern of murine *Plins* was similar to that of human tissues, with weak expression of *Plin4* in the mouse placenta. Hypoxia had a weak and statistically insignificant effect on the expression of relevant murine *Fatps* and *Plins* in both the placenta and the amnion (Fig. 2D–E). As a control, we showed reduced expression of placental transferrin receptor (*Tfrc*), a marker of hypoxic mouse placenta [37], [38].

**Figure 3:**
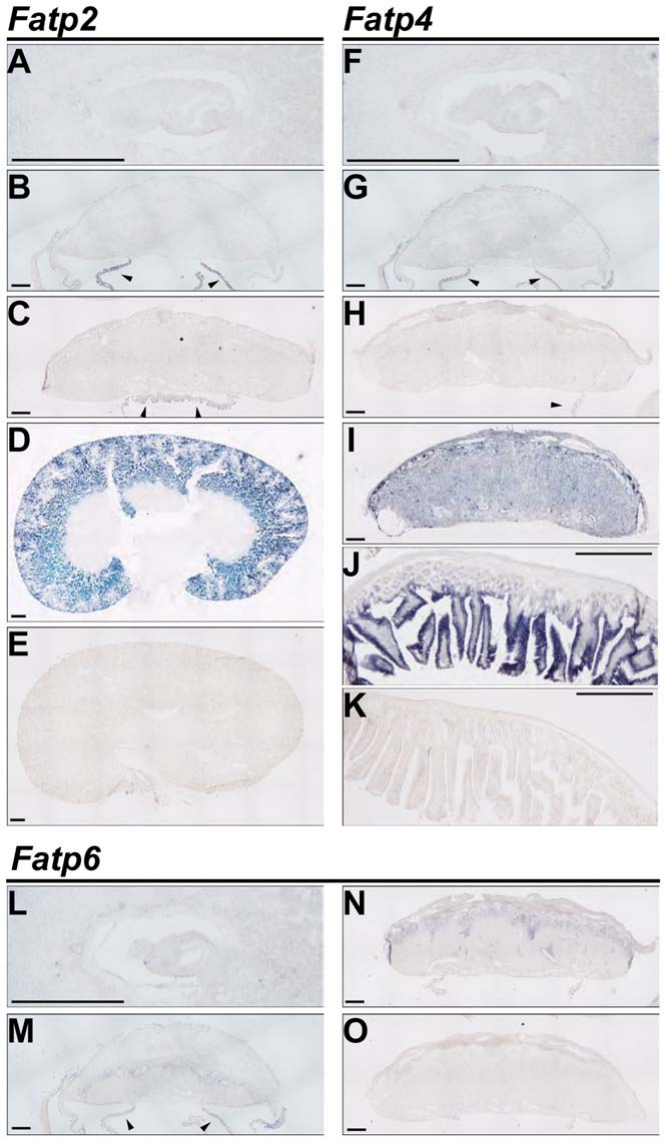
ISH for detection of Fatp2, Fatp4 and Fatp6 expression in the mouse placenta. *Fatp2* expression in the mouse embryo at E7.5 (A), placenta at E12.5 (B) and at E17.5 (C), and kidney (D). Arrowheads point to the amnion. *Fatp4* expression in mouse embryo at E7.5 (F), placenta at E12.5 (G) and at E17.5 (H), in the *Fatp4* transgenic placenta (I) and in the small intestine (J). *Fatp6* expression in mouse embryo at E7.5 (L), placenta at E12.5 (M) and at E17.5 (N). Negative controls using a sense probe are shown in E, K, O. Scale bars = 0.5 mm.

We used ISH to localize the expression of *Fatp2*, *Fatp4*, and *Fatp6* at E7.5 (prior to establishment of maternal-fetal trafficking), E12.5 (after maternal-fetal trafficking is initiated), and E17.5 (near-term placenta, Fig. 3). None of the three *Fatps* were definitively detected at E7.5. Using renal cortical expression of *Fatp2* as positive control [39], we found that *Fatp2* was expressed in the murine amnion but not in the placenta proper at E12.5 and E17.5 (Fig. 3, A–E). Similarly, *Fatp4* was expressed in amnion at E12.5, and exhibited weak, relatively diffuse placental expression at E12.5 and E17.5. This was confirmed using *Fatp4* expression in placentas derived from *Fatp4* transgenic mice (Fig. 3I), and using *Fatp4* expression in the intestinal villous epithelium as positive control (Fig. 3J). *Fatp6* was primarily detected in the spongiotrophoblast at E12.5 and E17.5, but not at E7.5 clearly (Fig. 3, L–O).

**Figure 4:**
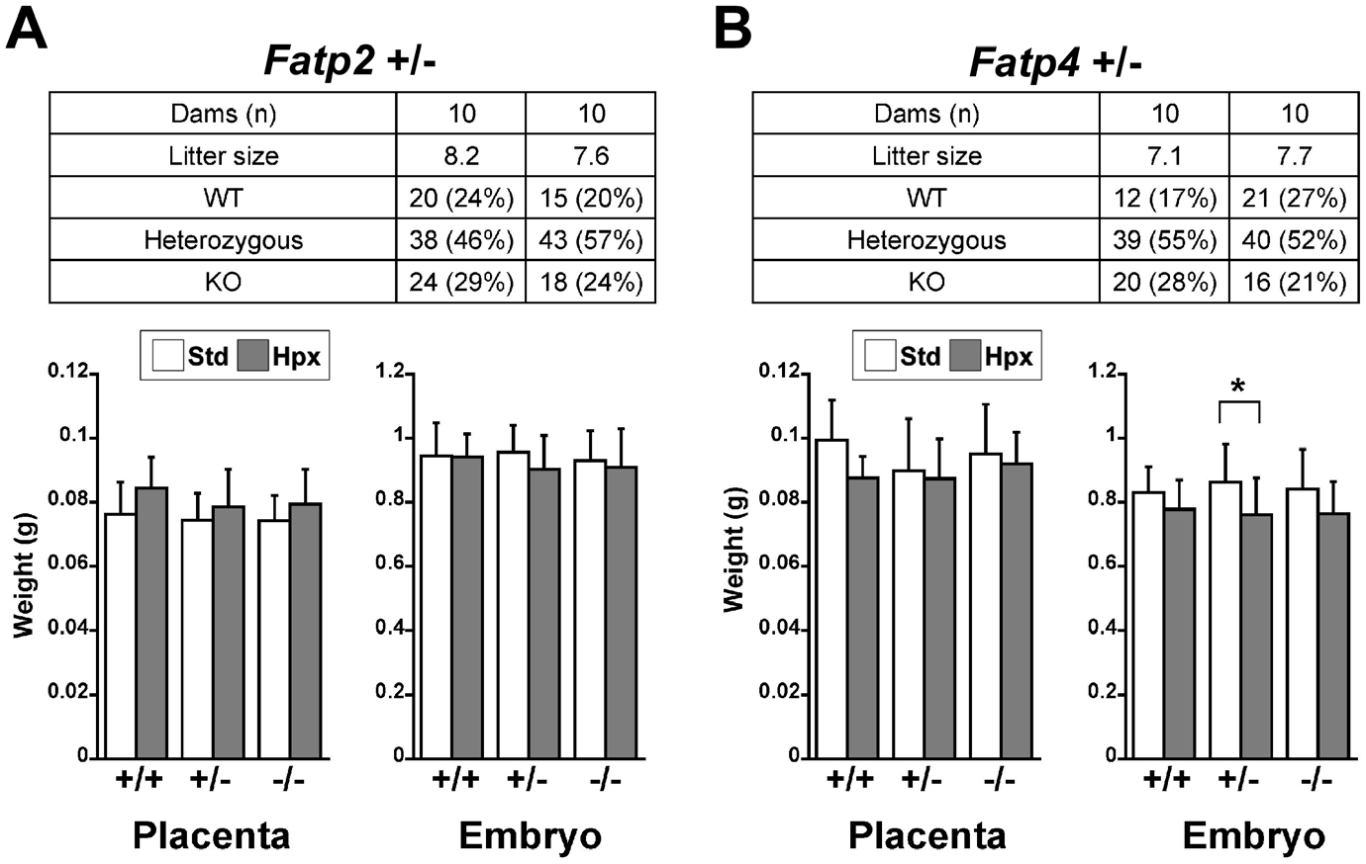
The feto-placental phenotype of Fatp2 and Fatp4 deficient mice. The litter size, genotype distribution, placental and embryo weight at E17.5 in standard or hypoxic conditions after cross-breeding of heterozygous pregnant mice. (A) *Fatp2* analysis, (B) *Fatp4* analysis. The graphs depict placenta and embryo weight. * denotes p<0.05.

**Figure 5:**
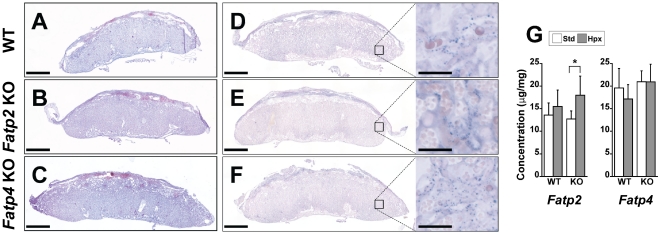
The effect of Fatp2 or Fatp4 deficiency on the levels of neutral lipids in the mouse placenta. Frozen sections stained with oil Red O (A–C) or with Sudan Black B (D–F) of wild type, *Fatp2* KO, or *Fatp4* KO at E17.5. Scale bars = 1 mm and 100 µm in larger panels and insets, respectively. (G) Triglyceride concentration of *Fatp2* wild type or KO (left panel), or *Fatp4* wild type or KO (right panel) in standard or hypoxic condition (n = 5 for each strain and each condition). * denotes p<0.05.

**Figure 6:**
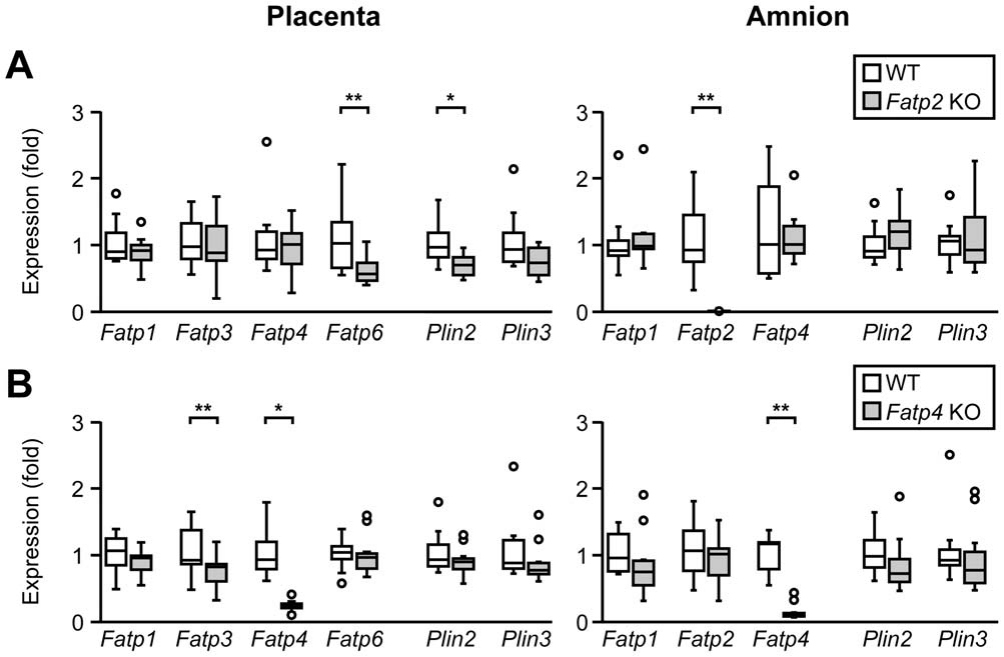
The effect of Fatp2 or Fatp4 deficiency on the expression of other Fatps or Plins in the mouse placenta. RT-qPCR of *Fatps*, *Plin2* and *Plin3* in the placenta or amnion, comparing expression in WT to KO placenta. Data include only relevant FATPs (A) *Fatp2* WT or KO (n = 12 each) (B) *Fatp4* WT or KO. * denotes p<0.05, ** denotes p<0.01.

We next sought to assess the impact of *Fatp2* or *Fatp4* deficiency on the murine placenta. Crossing heterozygous *Fatp2* or *Fatp4* males and females, we found that at E17.5 *Fatp2* or *Fatp4* deficient fetuses were indistinguishable from their wild type littermates in either standard or hypoxic conditions with respect to litter size, genotype distribution, placental and embryo weight (Fig. 4). In addition, placental histology was unchanged among the genotypes in either normal or hypoxic placenta (data not shown). Because FATPs play a role in cellular fatty acid uptake, we examined the accumulation of neutral fat, as well as triglyceride levels, in the placentas of wild type and *Fatp2* or *Fatp4* deficient mice. Oil Red O staining (Fig. 5A–C) and Sudan Black B staining (Fig. 5D–F) of placentas from all genotypes showed a similar pattern of diffuse fat staining, with more abundant fat in the decidua. This expression pattern was unchanged in hypoxic placentas (data not shown). Similarly, the concentration of placental triglycerides was also similar among the genotypes, with hypoxia causing a small yet significant increase in triglyceride concentration in the hypoxic *Fatp2* KO placenta (Fig. 5G). Lastly, we used quantitative RT-PCR to rule out the possibility that the expression of other *Fatps* might compensate for the reduced levels of *Fatp2* or *Fatp4*. As shown in Fig. 6, the expression of *Fatp2* or *Fatp4* was appropriately reduced in the respective placentas and amnion tissues, with reduced expression in placental *Fatp6* or *Fatp3* in the *Fatp2* and *Fatp4* KO placentas, respectively. Importantly, none of the *Fatps*, *Plin2* or *Plin3* exhibited a compensatory increase in expression.

## Discussion

The uptake, mobilization and efflux of fatty acids are critical for fetal growth, with fetuses of malnourished pregnant women being at risk for intrauterine growth restriction [10], [14], [40], [41]. Several observations regarding FATPs led us to interrogate the expression and function of placental FATPs: (a) hypoxic human trophoblasts accumulate neutral lipids with lipid droplets [35], and manuscript in preparation], (b) PPARg/RXR increase trophoblastic FATP1 and FATP4 expression, reduce FATP2 expression, and enhance trophoblast uptake of fatty acids and lipid droplet accumulation [42], and (c) inhibition of p38 (which mimics key aspects of PPARg deficiency) also up-regulates FATP2 and down-regulates FATP4 expression [42]. We found that among FATPs, *FATP2*, *FATP4* and *FATP6* are expressed in human trophoblasts, and that hypoxia enhanced *FATP2* and reduced *FATP4* expression levels. These data, which are opposite of the effect of PPARg/RXR signaling on FATP2 and FATP4, suggest that FATP2 and FATP4 play a role in trophoblast fatty acid trafficking. We therefore used pregnant mice to assess the function of FATP2 and FATP4 in the placenta *in vivo*. Although the human and mouse placenta share many structural, functional, and gene expression patterns [43], [44], there are marked morphological and functional differences between placentas of the two species, including differences in transport functions [45]–[47]. Whereas *FATP4* was expressed in both the human and murine placenta, the expression of murine *Fatp2* was restricted to the amnion. Unlike the expression changes in human trophoblastic *FATP2* and *FATP4* when cultured in hypoxic atmosphere, we found no difference in the expression of murine placental *Fatp2* and *Fatp4* between standard and hypoxic conditions *in vivo*.

We produced hypoxia during the latter part of mouse pregnancy using an O_2_ concentration of 12% for 6 days, which is similar to the level of hypoxia used by others and us [48], [49]. The most suitable degree of hypoxia for cultured PHT cells remains controversial. Low oxygen tension (pO_2_ of 15–20 mmHg) characterizes the early human placenta, before maternal blood begins to perfuse the intervillous space, with a rise to ∼55 mmHg after 12 weeks of pregnancy [50], [51]. Placental hypoxia is abnormal after that gestational age [52], [53]. Exposure of cultured third trimester trophoblasts to pO_2_<1%, as we chose in our experiments, is commonly used to model hypoperfusion-induced villous injury [54]–[56]. Notably, the differences between our *in vitro* analysis using PHT cells and intact mouse placentas likely reflect inter-species differences in expression patterns and functions of FATPs. Moreover, there is a clear dissimilarity between exposure to hypoxia *in vitro* and *in vivo*, where the response of purified cultured cells to extreme hypoxia might be different from that of intact tissue, which is exposed to marked, yet life-sustaining hypoxia.

Genetic ablation of murine *Fatp2* and *Fatp4* expression did not lead to any functional consequences with respect to feto-placental growth, and specifically, lipid accumulation. These data are consistent with those of Heinzer *et al* [57], which did not specifically focus on embryonic development, yet reported normal growth, behavior and activity of *Fatp2* KO mice. Moulson *et al* [58] reported that *Fatp4* KO mice died soon after birth, with shiny, tight, thick skin. This phenotype was reproduced by Herrmann *et al* [59], [60], who showed that epidermal-specific conditional *Fatp4* KO mice exhibited similar morphological abnormalities as embryos, indicating that the skin-related abnormalities of *Fatp4* KO fetuses reflect fetal maldevelopment and not placental dysfunction. In addition, Gimeno *et al* [61] demonstrated early embryonic lethality in *Fatp4* KO mice, possibly related to the expression of FATP4 in the extraembryonic endoderm. Interestingly, two other membrane-related fatty acid transporters, FABPpm and CD36 (FAT), are also expressed in the murine placenta, primarily in the labyrinth and junctional zone [17]. While a knockout mouse model for FABPpm has not been published, CD36 deficient mice do not exhibit a pregnancy or placenta –related phenotype [62].

Although we ruled out significant compensatory changes in placental *Fatp* expression in *Fatp2*- or *Fatp4*-deficient mice, we cannot rule out functional redundancy among fatty acid transporters. Such redundancy is unlikely, though, because the expression of the different *Fatp's* in the placenta is dissimilar. Furthermore, as noted above, the phenotype of *Fatp2*- and *Fatp4* -deficient mice in non-placental tissues does not overlap. Our data are the first to show the expression of *Fatp6*, hitherto known to be expressed in the human heart and bovine mammary tissue [12], [63], in human trophoblasts and mouse placentas, as well as in the female and male gonads. It is possible that FATP6 plays a role on fat trafficking in the placenta as well as in other organs, which may be redundant with the function of other FATPs.

While critical experiments that might have uncovered the role of FATPs in the human placenta cannot be ethically performed, our results underscore limitations in the use of animal models to inform human biology. Mutations in human FATP2 are currently unknown. Several mutations in human FATP4 are associated with ichthyosis prematurity syndrome, where preterm delivery is related to polyhydramnios, not a placental function [64], or with congenital verruciform hyperkeratosis [65]. New animal models as well as manipulation of FATP expression using *ex vivo* human samples might be necessary in order to fully analyze the role of FATPs in placental fat uptake and trafficking.

## Materials and Methods

### Primary human trophoblast (PHT) isolation and culture

Placental tissue samples were collected by the Obstetrical Specimen Procurement Unit at Magee-Womens Hospital of the University of Pittsburgh Medical Center. Collection was conducted under an approved exempt protocol by the Institutional Review Board of the University of Pittsburgh. Patients provided written consent for the use of de-identified, discarded tissues for research upon admittance to the Hospital. PHT cells were isolated from term human placentas (n = 5) and cultured as we previously described [55]. Cells were cultured in an atmosphere of 20% O_2_ with 5% CO_2_ at 37C as standard condition, or in O_2_<1% in a hermetically enclosed incubation chamber (Thermo Electron, Marietta, OH), where indicated [55]. Cells were harvested after 48 h and processed as detailed below.

### Mouse breeding, genotyping and exposure to hypoxia

Our experiments were conducted under protocol number 0806669-B4, which was approved by the Institutional Animal Care and Use Committee of University of Pittsburgh. *Fatp2* (*Slc27a2*tm1Kds) heterozygous C57Bl/6 mice harboring a targeted mutation in the *Fatp2* gene were generously provided by Dr. Kirby Smith (Johns Hopkins). *Fatp4* heterozygous (*Slc27a4*/wrfr) C57Bl/6 mice, which harbor a spontaneous transposon insertion in *Fatp4* gene, as well as the *Fatp4* overexpressing mouse, were previously described [66]. Timed matings were carried out by pairing heterozygous males and females for one night, with the morning after mating designated as embryonic day 0.5 (E0.5). Pregnancy was assumed based the presence of vaginal plug and a 10% weight gain on E10.5. Mice were kept under constant conditions until E11.5, were given a standard rodent chow and water *ad libitum*, and kept on a 12∶12 h light-dark cycle in room air. Delivery typically occurs on E19.5. Exposure to hypoxia, where relevant, was initiated on E11.5, when the mice were either exposed to FiO_2_ = 12% between E11.5 and E17.5 (hypoxia group) or normoxia at standard atmospheric conditions. For exposure to normobaric hypoxia we used a Polymer Hypoxic Glove Box with a Purge Airlock system with CO_2_ and O_2_ control indicators (Coy Laboratory Products, Grass Lake, MI), which is specifically designed for experiments in live rodents and regulates ambient temperature, humidity, and gas composition. Dams were euthanized by CO_2_ asphyxiation. Embryos and placentas were weighed, and immediately processed for further analysis (see below). Each set of analyses included ten pregnant mice, each carrying 6–10 embryos. Fetuses from uterine horns containing only one embryo were excluded from the final analysis. Genomic DNA was extracted from embryo tails by the alkaline lysis and boiling method [67] and genotyped using standard PCR, as previously described [34], [58].

### Histological Analysis

For oil Red O staining, 4% PFA-fixed samples were immersed in 10%, then 20% sucrose in PBS, followed by OCT embedding. Sections were cut using a cryostat (Cryotome FSE, Thermo Scientific, Wilmington, DE) at 7 µm thickness, then stained with oil Red O (Sigma) [68] and counter-stained with hematoxylin or stained with Sudan Black B (Sigma) and counter-stained with nuclear fast red.

For detection of *Fatp2*, *Fatp4* and *Fatp6* by ISH we used digoxigenin-labeled cRNA probes, synthesized using digoxigenin RNA labeling kit (Roche, Basel, Switzerland). Cryosections (10 µm) of the OCT-embedded placentas were rehydrated in PBS, digested with proteinase K (10 µg/ml, 5 min at 37 C), treated with 0.2 N HCl for 10 min at RT, acetylated (0.25% acetic anhydride in TEA for 10 min at RT), and then hybridized with cRNA probes overnight at 60 C. Slides were washed four times with 4×SSC, digested with RNaseA (5 µg/ml) for 15 min at 37 C, and washed twice with 0.5×SSC for 15 min at 60 C. Slides were blocked using 1% blocking reagent (Roche) in maleic acid buffer (MAB), followed by incubation with anti-DIG-AP antibody (0.5 U/ml) for 2 h at RT, washed with MABT (MAB with 0.2% Tween20), and then reacted with BM purple (Roche) with 1 mM levamisole overnight. The sections were examined using Nikon 90i microscope (Nikon, Tokyo, Japan) equipped with DS-Ri1 CCD camera (Nikon).

### Standard and quantitative RT-PCR

Total RNA was extracted from PHT cells or from diverse tissues of eight weeks old mice using TriReagent (MRC, Cincinnati, OH) according to the manufacturer's instructions. Some of the analyses were also performed using Human Total RNA Survey Panel (Ambion, Austin, CA). RNA samples were treated with DNaseI using a Turbo DNA-free Kit (Ambion). Complementary DNA (cDNA) was synthesized from 1 µg of total RNA in 20 µl of reaction mixture using High Capacity RNA-to-cDNA Master Mix (Applied Biosystems, Foster City, CA). Synthesized cDNA samples were diluted 1∶5 in DEPC-treated H_2_O. RNA quality was assessed by 260/280 and 260/230 absorbance ratio using NanoDrop (Thermo). Standard RT-PCR was performed using KOD Xtreme DNA polymerase (EMD, Gibbstown, NJ) with 2 µl cDNA per 20 µl reaction volume, with amplification in a Veriti thermal cycler (Applied Biosystems) using the following conditions: 94 C for 2 min, 35 cycles at 98 C for 10 sec, 60 C for 30 sec, and 68 C at 30 sec. PCR products were electrophoresed on a 2% TAE agarose gel and DNA detected using ethidium bromide. Quantitative RT-PCR was carried out in duplicates using 384 well plates with 2 µl of cDNA per 10 µl of reaction mixture using SYBR Green PCR master mix (Applied Biosystems). A total of 8–12 cDNA from mouse samples and five cDNAs from human samples were used for each analysis. PCR was carried out in Geneamp 7900 (Applied Biosystems). The specificity of amplification was confirmed using a dissociation curve of the PCR product. Detection of YWHAZ for human [69] or L32 for mouse [70] was used as normalization control. The relative expression change was calculated using the ΔΔC_t_ method [71]. All primers used in this study are listed in Table S1.

### Western blot analysis

Proteins for western blot were prepared from cells using lysis buffer, and by homogenization in lysis buffer for tissues, as we previously described [35]. Protein lysates were electrophoresed using 7.5% sodium dodecyl sulfate (SDS)-polyacrylamide gel at 180 V for 1 h, then transferred to polyvinylidene difluoride membranes (Biorad, Hercules, CA) at 23 V for overnight. After blocking with 5% non-fat dried milk in TBST, the membranes were incubated overnight with rabbit anti-FATP2, FATP4 antibodies as previously described [30] or mouse anti-actin, Millipore, Bedford, MA) antibodies at 4 C. After washing with TBST, the membranes were incubated with goat anti-rabbit IgG peroxidase conjugated (Santa Cruz Biotech, Santa Cruz, CA) and donkey anti-mouse IgG peroxidase conjgated (Santa Cruz) for 2 h at RT. Detection was performed with Western Lightning ECL kit (Perkin Elmer).

### Triglyceride Assay

Lipids were extracted from each murine placenta by the Folch method [72], and triglyceride concentration determined using a triglyceride spectrophotometer assay kit (Cayman Chemical, Ann Arbor, MI), detected using a VersaMax microplate reader (Molecular Devices, Sunnyvale, CA). Five placental fragments, each 10–20 µg, were used for analysis, and normalized by weight.

### Statistics

Statistical analysis was performed using analysis of variance (ANOVA) with Bonferroni post hoc test for multiple comparisons of placental and embryo weight, and by Mann-Whitney test for RT-qPCR and triglyceride assay using Dr SPSS II for Windows (SPSS, Chicago, IL). Significance was determined at p<0.05.

## Supporting Information

Table S1 **Primers used in standard and RT-qPCR.**
(DOC)

## References

[1] Hornstra G, Al MD, van Houwelingen AC, Foreman-van Drongelen MM (1995). Essential fatty acids in pregnancy and early human development.. Eur J Obstet Gynecol Reprod Biol.

[2] Dutta-Roy AK (2000). Transport mechanisms for long-chain polyunsaturated fatty acids in the human placenta.. Am J Clin Nutr.

[3] Haggarty P (2002). Placental regulation of fatty acid delivery and its effect on fetal growth–a review.. Placenta.

[4] Neuringer M, Connor WE, Lin DS, Barstad L, Luck S (1986). Biochemical and functional effects of prenatal and postnatal omega 3 fatty acid deficiency on retina and brain in rhesus monkeys.. Proc Natl Acad Sci USA.

[5] Uauy R, Treen M, Hoffman DR (1989). Essential fatty acid metabolism and requirements during development.. Semin Perinatol.

[6] Larque E, Demmelmair H, Koletzko B (2002). Perinatal supply and metabolism of long-chain polyunsaturated fatty acids: importance for the early development of the nervous system.. Ann N Y Acad Sci.

[7] Lauritzen L, Hansen HS, Jorgensen MH, Michaelsen KF (2001). The essentiality of long chain n-3 fatty acids in relation to development and function of the brain and retina.. Prog Lipid Res.

[8] Herrera E, Amusquivar E (2000). Lipid metabolism in the fetus and the newborn.. Diabetes Metab Res Rev.

[9] Knipp GT, Audus KL, Soares MJ (1999). Nutrient transport across the placenta.. Adv Drug Deliv Rev.

[10] Belkacemi L, Nelson DM, Desai M, Ross MG (2010). Maternal undernutrition influences placental-fetal development.. Biol Reprod.

[11] Doege H, Stahl A (2006). Protein-mediated fatty acid uptake: novel insights from in vivo models.. Physiology (Bethesda).

[12] Duttaroy AK (2009). Transport of fatty acids across the human placenta: a review.. Prog Lipid Res.

[13] Bonet B, Brunzell JD, Gown AM, Knopp RH (1992). Metabolism of very-low-density lipoprotein triglyceride by human placental cells: the role of lipoprotein lipase.. Metabolism.

[14] Magnusson AL, Waterman IJ, Wennergren M, Jansson T, Powell TL (2004). Triglyceride hydrolase activities and expression of fatty acid binding proteins in the human placenta in pregnancies complicated by intrauterine growth restriction and diabetes.. J Clin Endocrinol Metab.

[15] Magnusson-Olsson AL, Lager S, Jacobsson B, Jansson T, Powell TL (2007). Effect of maternal triglycerides and free fatty acids on placental LPL in cultured primary trophoblast cells and in a case of maternal LPL deficiency.. Am J Physiol Endocrinol Metab.

[16] Dutta-Roy AK (2000). Cellular uptake of long-chain fatty acids: role of membrane-associated fatty-acid-binding/transport proteins.. Cell Mol Life Sci.

[17] Knipp GT, Liu B, Audus KL, Fujii H, Ono T (2000). Fatty acid transport regulatory proteins in the developing rat placenta and in trophoblast cell culture models.. Placenta.

[18] Daoud G, Simoneau L, Masse A, Rassart E, Lafond J (2005). Expression of cFABP and PPAR in trophoblast cells: effect of PPAR ligands on linoleic acid uptake and differentiation.. Biochim Biophys Acta.

[19] Ducharme NA, Bickel PE (2008). Lipid droplets in lipogenesis and lipolysis.. Endocrinology.

[20] Farese RV, Walther TC (2009). Lipid droplets finally get a little R-E-S-P-E-C-T.. Cell.

[21] Martin S, Parton RG (2006). Lipid droplets: a unified view of a dynamic organelle.. Nat Rev Mol Cell Biol.

[22] Brown DA (2001). Lipid droplets: proteins floating on a pool of fat.. Curr Biol.

[23] Miura S, Gan JW, Brzostowski J, Parisi MJ, Schultz CJ (2002). Functional conservation for lipid storage droplet association among Perilipin, ADRP, and TIP47 (PAT)-related proteins in mammals, Drosophila, and Dictyostelium.. J Biol Chem.

[24] Brasaemle DL (2007). Thematic review series: adipocyte biology. The perilipin family of structural lipid droplet proteins: stabilization of lipid droplets and control of lipolysis.. J Lipid Res.

[25] Kimmel AR, Brasaemle DL, McAndrews-Hill M, Sztalryd C, Londos C (2010). Adoption of PERILIPIN as a unifying nomenclature for the mammalian PAT-family of intracellular lipid storage droplet proteins.. J Lipid Res.

[26] Hirsch D, Stahl A, Lodish HF (1998). A family of fatty acid transporters conserved from mycobacterium to man.. Proc Natl Acad Sci USA.

[27] Schaffer JE (2002). Fatty acid transport: the roads taken.. Am J Physiol Endocrinol Metab.

[28] Stahl A (2004). A current review of fatty acid transport proteins (SLC27).. Pflugers Arch.

[29] Gimeno RE, Ortegon AM, Patel S, Punreddy S, Ge P (2003). Characterization of a heart-specific fatty acid transport protein.. J Biol Chem.

[30] Stahl A, Hirsch DJ, Gimeno RE, Punreddy S, Ge P (1999). Identification of the major intestinal fatty acid transport protein.. Mol Cell.

[31] Stahl A, Gimeno RE, Tartaglia LA, Lodish HF (2001). Fatty acid transport proteins: a current view of a growing family.. Trends Endocrinol Metab.

[32] Hall AM, Wiczer BM, Herrmann T, Stremmel W, Bernlohr DA (2005). Enzymatic properties of purified murine fatty acid transport protein 4 and analysis of acyl-CoA synthetase activities in tissues from FATP4 null mice.. J Biol Chem.

[33] Herrmann T, Buchkremer F, Gosch I, Hall AM, Bernlohr DA (2001). Mouse fatty acid transport protein 4 (FATP4): characterization of the gene and functional assessment as a very long chain acyl-CoA synthetase.. Gene.

[34] Heinzer AK, Watkins PA, Lu JF, Kemp S, Moser AB (2003). A very long-chain acyl-CoA synthetase-deficient mouse and its relevance to X-linked adrenoleukodystrophy.. Hum Mol Genet.

[35] Biron-Shental T, Schaiff WT, Ratajczak CK, Bildirici I, Nelson DM (2007). Hypoxia regulates the expression of fatty acid-binding proteins in primary term human trophoblasts.. Am J Obstet Gynecol.

[36] Roh CR, Budhraja V, Kim HS, Nelson DM, Sadovsky Y (2005). Microarray-based identification of differentially expressed genes in hypoxic term human trophoblasts and in placental villi of pregnancies with growth restricted fetuses.. Placenta.

[37] Mando C, Tabano S, Colapietro P, Pileri P, Colleoni F (2010). Transferrin receptor gene and protein expression and localization in human IUGR and normal term placentas.. Placenta.

[38] Gheorghe CP, Mohan S, Oberg KC, Longo LD (2007). Gene expression patterns in the hypoxic murine placenta: a role in epigenesis?. Reprod Sci.

[39] Johnson AC, Stahl A, Zager RA (2005). Triglyceride accumulation in injured renal tubular cells: alterations in both synthetic and catabolic pathways.. Kidney Int.

[40] Araya J, Soto C, Aguilera AM, Bosco C, Monlina R (1995). [Modification of the lipid profile of human placenta by moderate maternal undernutrition].. Rev Med Chil.

[41] Cetin I, Giovannini N, Alvino G, Agostoni C, Riva E (2002). Intrauterine growth restriction is associated with changes in polyunsaturated fatty acid fetal-maternal relationships.. Pediatr Res.

[42] Schaiff WT, Bildirici I, Cheong M, Chern PL, Nelson DM (2005). Peroxisome proliferator-activated receptor-gamma and retinoid X receptor signaling regulate fatty acid uptake by primary human placental trophoblasts.. J Clin Endocrinol Metab.

[43] Georgiades P, Ferguson-Smith AC, Burton GJ (2002). Comparative developmental anatomy of the murine and human definitive placentae.. Placenta.

[44] Cox B, Kotlyar M, Evangelou AI, Ignatchenko V, Ignatchenko A (2009). Comparative systems biology of human and mouse as a tool to guide the modeling of human placental pathology.. Mol Syst Biol.

[45] Takizawa T, Anderson CL, Robinson JM (2005). A novel Fc gamma R-defined, IgG-containing organelle in placental endothelium.. J Immunol.

[46] Mohanty S, Kim J, Ganesan LP, Phillips GS, Hua K (2010). IgG is transported across the mouse yolk sac independently of FcgammaRIIb.. J Reprod Immunol.

[47] Kim J, Mohanty S, Ganesan LP, Hua K, Jarjoura D (2009). FcRn in the yolk sac endoderm of mouse is required for IgG transport to fetus.. J Immunol.

[48] Carter AM (2007). Animal models of human placentation–a review.. Placenta.

[49] Vuguin PM (2007). Animal models for small for gestational age and fetal programming of adult disease.. Horm Res.

[50] Burton GJ, Jauniaux E, Watson AL (1999). Maternal arterial connections to the placental intervillous space during the first trimester of human pregnancy: the Boyd collection revisited.. Am J Obstet Gynecol.

[51] Rodesch F, Simon P, Donner C, Jauniaux E (1992). Oxygen measurements in endometrial and trophoblastic tissues during early pregnancy.. Obstet Gynecol.

[52] Fox H (1970). Effect of hypoxia on trophoblast in organ culture. A morphologic and autoradiographic study.. Am J Obstet Gynecol.

[53] Cetin I, Alvino G (2009). Intrauterine growth restriction: implications for placental metabolism and transport.. A review. Placenta.

[54] Alsat E, Wyplosz P, Malassine A, Guibourdenche J, Porquet D (1996). Hypoxia impairs cell fusion and differentiation process in human cytotrophoblast, *in vitro*.. J Cell Physiol.

[55] Mouillet JF, Chu T, Nelson DM, Mishima T, Sadovsky Y (2010). MiR-205 silences MED1 in hypoxic primary human trophoblasts.. FASEB J.

[56] Peltier MR, Gurzenda EM, Murthy A, Chawala K, Lerner V (2011). Can oxygen Tension Contribute to an Abnormal Placental Cytokine Milieu?. Am J Reprod Immunol.

[57] Heinzer AK, McGuinness MC, Lu JF, Stine OC, Wei H (2003). Mouse models and genetic modifiers in X-linked adrenoleukodystrophy.. Adv Exp Med Biol.

[58] Moulson CL, Martin DR, Lugus JJ, Schaffer JE, Lind AC (2003). Cloning of wrinkle-free, a previously uncharacterized mouse mutation, reveals crucial roles for fatty acid transport protein 4 in skin and hair development.. Proc Natl Acad Sci USA.

[59] Herrmann T, van der Hoeven F, Grone HJ, Stewart AF, Langbein L (2003). Mice with targeted disruption of the fatty acid transport protein 4 (Fatp 4, Slc27a4) gene show features of lethal restrictive dermopathy.. J Cell Biol.

[60] Herrmann T, Grone HJ, Langbein L, Kaiser I, Gosch I (2005). Disturbed epidermal structure in mice with temporally controlled fatp4 deficiency.. J Invest Dermatol.

[61] Gimeno RE, Hirsch DJ, Punreddy S, Sun Y, Ortegon AM (2003). Targeted deletion of fatty acid transport protein-4 results in early embryonic lethality.. J Biol Chem.

[62] Febbraio M, Abumrad NA, Hajjar DP, Sharma K, Cheng W (1999). A null mutation in murine CD36 reveals an important role in fatty acid and lipoprotein metabolism.. J Biol Chem.

[63] Bionaz M, Loor JJ (2008). ACSL1, AGPAT6, FABP3, LPIN1, and SLC27A6 are the most abundant isoforms in bovine mammary tissue and their expression is affected by stage of lactation.. J Nutr.

[64] Klar J, Schweiger M, Zimmerman R, Zechner R, Li H (2009). Mutations in the fatty acid transport protein 4 gene cause the ichthyosis prematurity syndrome.. Am J Hum Genet.

[65] Morice-Picard F, Leaute-Labreze C, Decor A, Boralevi F, Lacombe D (2010). A novel mutation in the fatty acid transport protein 4 gene in a patient initially described as affected by self-healing congenital verruciform hyperkeratosis.. Am J Med Genet A.

[66] Moulson CL, Lin MH, White JM, Newberry EP, Davidson NO (2007). Keratinocyte-specific expression of fatty acid transport protein 4 rescues the wrinkle-free phenotype in Slc27a4/Fatp4 mutant mice.. J Biol Chem.

[67] Hanley T, Merlie JP (1991). Transgene detection in unpurified mouse tail DNA by polymerase chain reaction.. Biotechniques.

[68] Koopman R, Schaart G, Hesselink MK (2001). Optimisation of oil red O staining permits combination with immunofluorescence and automated quantification of lipids.. Histochem Cell Biol.

[69] Meller M, Vadachkoria S, Luthy DA, Williams MA (2005). Evaluation of housekeeping genes in placental comparative expression studies.. Placenta.

[70] Maity A, Solomon D (2000). Both increased stability and transcription contribute to the induction of the urokinase plasminogen activator receptor (uPAR) message by hypoxia.. Exp Cell Res.

[71] Livak KJ, Schmittgen TD (2001). Analysis of relative gene expression data using real-time quantitative PCR and the 2(−Delta Delta C(T)) Method.. Methods.

[72] Folch J, Lees M, Sloane Stanley GH (1957). A simple method for the isolation and purification of total lipides from animal tissues.. J Biol Chem.

