# Supplementary material for: The Expression and Function of Fatty Acid Transport Protein-2 and -4 in the Murine Placenta
Source: PLoS One. 2011 Oct 20;6(10):e25865. doi: 10.1371/journal.pone.0025865 (PMC3197585; doi:10.1371/journal.pone.0025865)
Supplement: Table S1 — Primers used in standard and RT-qPCR. (DOC) [file pone.0025865.s001.doc]

**Supplementary Table 1.** Primers used in standard and RT-qPCR.

| **Human Primers (Regular RT-PCR)** | | | | | | | | **Human Primers (Real Time PCR)** | | |
| --- | --- | --- | --- | --- | --- | --- | --- | --- | --- | --- |
| **Gene** | **Accession no.** | **F/R** | **Position** | | **Size** | | **Sequence** | **Position** | **Size** | **Sequence** |
| *FATP1* | NM_198580.1 | F | 632-651 | | 316 | | GCATCTGGGGAAAAGTTTGA | 2952-2973 | 114 | CCGGAATTGACTGTGACCACTT |
|  |  | R | 947-928 | |  | | GATGATGTTTCCTGCCGAGT | 3065-3048 |  | CACGCAGTGCAGGGTTCA |
| *FATP2* | NM_003645.3 | F | 702-721 | | 403 | | TGTCGCCAGAACTACAAGCA^1^ | 1829-1852 | 80 | CTTTCAGCACATTGCTGATTACCT |
|  |  | R | 1104-1085 | |  | | TTAGTCCGCAAGGCAAGAGT^2^ | 1908-1884 |  | CAGTGATCTCAATGGTGTCCTGTAT |
| *FATP3* | NM_024330.1 | F | 1426-1445 | | 433 | | ATACCTGGGAGCGTTTTGTG | 2098-2122 | 83 | CAGAGACCTTCAAACAGCAGAAAGT |
|  |  | R | 1858-1839 | |  | | TCCACCTGAAGGTGTCTCC | 2180-2158 |  | CAGAACGTACAGTGGGTCAGACA |
| *FATP4* | NM_005094.2 | F | 1730-1749 | | 331 | | CCAGGCCTACCTTACTGGTG | 470-489 | 71 | GAAGGCAAAGGTGCGACAGT |
|  |  | R | 2060-2041 | |  | | TCCTGTTTTGTGCAGCTCAG | 540-522 |  | GCCGAACGGTAGAGGCAAA |
| *FATP5* | NM_012254.2 | F | 1159-1178 | | 420 | | TAGATCTCGGAGCCACCTGT |  |  |  |
|  |  | R | 1578-1559 | |  | | CTAGCCCTACAGGGATGCAG |  |  |  |
| *FATP6* | NM_014031.3 | F | 1229-1248 | | 312 | | GTGTTGAGTTGGGTGCCACT | 1007-1024 | 65 | GCGTGGTGGCCTTTCTCA |
|  |  | R | 1540-1521 | |  | | TCCCAATTGCTCCAATTCTC | 1071-1055 |  | ACAGGCGCGGATGCAAT |
| *PLIN1* | NM_002666.4 | F | 651-670 | | 504 | | CCAGAGACACTGCGGAATTT |  |  |  |
|  |  | R | 1154-1135 | |  | | GGGCTGCTACCTCACTGAAC |  |  |  |
| *PLIN2* | NM_001122.2 | F | 202-221 | | 514 | | CACAACCGAGTGTGGTGACT |  |  |  |
|  |  | R | 715-696 | |  | | GCATTTTCTACGCCACTGCT |  |  |  |
| *PLIN3* | NM_005817.4 | F | 209-228 | | 377 | | TATGCCTCCACCAAGGAGAG |  |  |  |
|  |  | R | 585-566 | |  | | CCGGTCACTACGGACTTTGT |  |  |  |
| *PLIN4* | NM_001080400.1 | F | 3168-3187 | | 501 | | CAGGACCACAGACAATGGTG |  |  |  |
|  |  | R | 3668-3649 | |  | | TTTTCAATCAGCCTGAAGCA |  |  |  |
| *PLIN5* | NM_001013706.2 | F | 176-195 | | 538 | | TGTTTACAGTGCAGCCAAGG |  |  |  |
|  |  | R | 713-694 | |  | | CAGTTTCCCCACAGAGTGCT |  |  |  |
| *YWHAZ* | NM_001135702.1 | F |  | |  | |  | 1184-1207 | 94 | ACTTTTGGTACATTGTGGCTTCAA |
|  |  | R |  | |  | |  | 1277-1258 |  | CCGCCAGGACAAACCAGTAT |
| *GAPDH* | NM_002046.3 | F | 122-141 | | 434 | | GAGTCAACGGATTTGGTCGT |  |  |  |
|  |  | R | 555-536 | |  | | GGAGGCATTGCTGATGATCT |  |  |  |
| **Mouse Primers (Regular RT-PCR)** | | | | | | | | **Mouse Primers (Real Time PCR)** | | |
| *Fatp1* | NM_011977.3 | F | | 1080-1099 | 552 | ACCACTCTGCAGGGAACATC | | 211-229 | 65 | GGCTCCTGGAGCAGGAACA |
|  |  | R | | 1631-1612 |  | TCTTCTTGTTGGTGGCACTG | | 275-256 |  | ACGGAAGTCCCAGAAACCAA |
| *Fatp2* | NM_011978.2 | F | | 600-619 | 390 | GCCTCCCCAGATCTACAAGA | | 810-829 | 180 | GGAACCACAGGTCTTCCAAA |
|  |  | R | | 989-970 |  | TAAAGTAGCCCCAACCACGA | | 989-970 |  | TAAAGTAGCCCCAACCACGA |
| *Fatp3* | NM_011988.1 | F | | 1181-1200 | 561 | CCAGAAACACAGGGTGACAGTGT | | 1899-1921 | 75 | CAGCTCTACAGCCATGTTTCTGA |
|  |  | R | | 1741-1722 |  | TTCCACCTGAAGGTGTCTCC | | 1973-1952 |  | CAAAGATTCCTGGAGCCTGAGA |
| *Fatp4* | NM_011989.4 | F | | 567-586 | 608 | ATTTTCGAGGGCACAGACAC | | 1085-1107 | 90 | ACGATGTTTCCTGCTGAGTGGTA |
|  |  | R | | 1174-1152 |  | ACGATGTTTCCTGCTGAGTGGTA | | 1174-1152 |  | CTCTCCGACCTGCCACAGA |
| *Fatp5* | NM_009512.2 | F | | 1500-1519 | 530 | TTCGAATGCTGACTCCCTTT | |  |  |  |
|  |  | R | | 2029-2010 |  | GGAATCCTGGATACGGATGA | |  |  |  |
| *Fatp6* | NM_001081072.1 | F | | 1179-1198 | 572 | TGCAAACAGCCTCAGAGAGA | | 2311-2329 | 77 | GGCTTGAGGATGCCGCTTA |
|  |  | R | | 1750-1731 |  | GCTTCCTGGATGAAGTCCAA | | 2387-2363 |  | GTACTCTGGGCTCATGCTATGAAGT |
| *Plin1* | NM_175640.2 | F | | 902-922 | 408 | ATGAGAGCCATGACGACCAGA | |  |  |  |
|  |  | R | | 1309-1287 |  | CTGCTGAAGGGTTATCGATGTCT | |  |  |  |
| *Plin2* | NM_007408.3 | F | | 716-735 | 528 | CTCAGGAGGAGCTGGAGATG | |  |  |  |
|  |  | R | | 1243-1224 |  | CGCCATCGGACACTTCCTTA | |  |  |  |
| *Plin3* | NM_025836.3 | F | | 116-138 | 398 | CTGCTATGGAGGAACCTGTTGTG | |  |  |  |
|  |  | R | | 513-490 |  | TGTTTCCTTCGCACTAGACACTGA | |  |  |  |
| *Plin4* | NM_020568.3 | F | | 4182-4202 | 408 | GTCTTCGGAGGCAGGTAGTGA | |  |  |  |
|  |  | R | | 4589-5468 |  | GGACGCGTGATGCTTCTTTACT | |  |  |  |
| *Plin5* | NM_025874.3 | F | | 492-513 | 280 | CATCAGACATGGTGGTGACATC | |  |  |  |
|  |  | R | | 771-751 |  | GACCCCAGACGCACAAAGTAG | |  |  |  |
| *L32* | NN_172086.2 | F | |  |  |  | | 67-87 | 102 | CCTCTGGTGAAGCCCAAGATC |
|  |  | R | |  |  |  | | 168-150 |  | TCTGGGTTTCCGCCAGTTT |
| *Gapdh* | NM_008084.2 | F | | 150-169 | 401 | CCCTTCATTGACCTCAACTA | |  |  |  |
|  |  | R | | 550-531 |  | CCAAAGTTGTCATGGATGAC | |  |  |  |
| *Trfc* | NM_011638 | F | |  |  |  | | 1495-1516 | 152 | GTTTCTGCCAGCCCCTTATTAT^3^ |
|  |  | R | |  |  |  | | 1646-1626 |  | GCAAGGAAAGGATATGCAGCA |

^1^Variant 1: 403bp

^2^Variant 2: 270bp

^3^ PrimerBank: 11596855a1
